# Dual role of HO-1 in mediating antiviral immune responses and mitigating excessive inflammatory damage during influenza virus infection

**DOI:** 10.1016/j.isci.2025.112116

**Published:** 2025-02-26

**Authors:** Linlin Ma, Peng Zhang, Xingqiong Li, Baihe Sun, Yuhuan Li, Jiandong Jiang

**Affiliations:** 1 Shanghai University of Medicine & Health Sciences Affiliated Zhoupu Hospital, Shanghai, China; 2 CAMS Key Laboratory of Antiviral Drug Research, Institute of Medicinal Biotechnology, Chinese Academy of Medical Sciences and Peking Union Medical College, Beijing, China; 3 Beijing Key Laboratory of Antimicrobial Agents, Institute of Medicinal Biotechnology, Chinese Academy of Medical Sciences and Peking Union Medical College, Beijing, China; 4 NHC Key Laboratory of Biotechnology for Microbial Drugs, Institute of Medicinal Biotechnology, Chinese Academy of Medical Sciences and Peking Union Medical College, Beijing, China

**Keywords:** virology, biochemistry, immunology

## Abstract

Influenza A virus (IAV) remains a global health threat, with severe cases causing high morbidity and mortality. Type I interferons (IFN-α/β) are crucial for early innate immunity to IAV but can drive immunopathology. This study investigates the role of heme oxygenase-1 (HO-1), a stress-responsive protein with anti-inflammatory properties, in modulating the immune response to IAV. Lung-tropic adeno-associated virus (AAV)-mediated HO-1 overexpression reduces lung damage by limiting immune cells infiltration, including plasmacytoid dendritic cells and classical monocytes, while promoting regulatory T cells (Tregs) and nonclassical monocytes. Additionally, HO-1 increases macrophage populations, enhancing antiviral responses via IFN pathways. Consistent with this, HO-1 knockout mice experience more severe infections, and HO-1 recruit inhibited IAV replication and alleviated pulmonary inflammation. In addition, compared with wide-type, the catalytically inactive mutation (H25A) impairs HO-1’s anti-inflammatory function. These findings underscore HO-1’s critical role in balancing antiviral immunity and inflammation, positioning it as a potential therapeutic target for severe influenza.

## Highlights

• HO-1 balances antiviral immunity and excessive lung inflammation in IAV infection
• HO-1 reduces pDC and classical monocyte, enhances Tregs and nonclassical monocytes
• HO-1 promotes macrophage populations, boosting antiviral immune responses
• Catalytically inactive H25A mutation impairs the anti-inflammatory function of HO-1

## Graphical abstract

**Figure:**
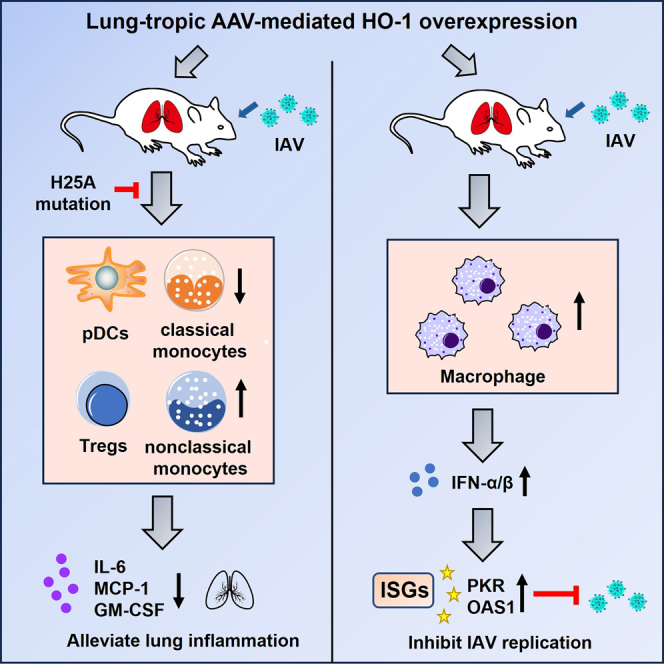

## Introduction

Influenza ranks among the most lethal infectious diseases in human history, with the 1918 H1N1 “Spanish flu” pandemic standing out as the deadliest, affecting over 200 countries and causing nearly 100 million deaths. Despite the growing availability of antiviral drugs and vaccines, severe influenza caused by the influenza A virus (IAV) continues to pose a significant threat to human health, with seasonal influenza responsible for approximately 500,000 deaths annually.^1^^,^^2^

The innate immune system serves as the first line of defense against influenza virus infection. Upon entry into respiratory epithelial cells, influenza viruses are recognized by intracellular pattern recognition receptors (PRRs) such as RIG-1 and TLR7, which detect the infection and replication process, thereby triggering the innate immune response and initiating antiviral activity.^3^ Type I interferons (IFNs), specifically IFN-α/β, play a pivotal role in the early stages of antiviral immune responses. IFN-α/β can inhibit viral replication by promoting apoptosis in infected cells and inducing the expression of IFN-stimulated genes (ISGs) such as 2′,5′-oligoadenylate synthetase 1 (OAS1), protein kinase R (PKR), and interferon-induced transmembrane protein 3 (IFITM3).^4^ Our previous research demonstrated that the sesquiterpene derivative YZH-106 inhibits influenza virus replication in RAW264.7 cells by inducing IFN-α/β.^5^ Consistent with the *in vitro* antiviral effects of IFN-α/β, exogenous IFN-α has also been shown to suppress the replication of H1N1 and H5N1 influenza viruses in animal models.^6^^,^^7^

However, in addition to its antiviral effects, IFN-α/β can also contribute to inflammatory damage and apoptosis in lung epithelial cells. A recent study found that in influenza virus-infected mice, elevated levels of IFN-α/β were correlated with increased lung injury and higher mortality rates. This effect is associated not only with enhanced infiltration of natural killer (NK) cells and inflammatory neutrophils into the lungs but also with DR5-TRAIL-mediated apoptosis of epithelial cells.^8^ Similarly, another study reported that macrophage-derived IFN-β induced apoptosis in alveolar epithelial cells.^9^ These findings indicate that while type I IFN-mediated innate immune activation is essential for controlling viral replication, it also inflicts damage on the infected tissues. The ability to balance these effects, controlling viral replication effectively without inducing severe inflammatory damage, likely influences the severity of influenza symptoms in patients. However, the specific factors and mechanisms that regulate and balance this process during influenza infection remain to be fully elucidated.

Heme oxygenase-1 (HO-1) is a stress-responsive protein with a catalytic domain that plays a role in heme metabolism, exhibiting anti-inflammatory and anti-apoptotic properties.^10^ Additionally, research has shown that HO-1 forms a complex with interferon regulatory factor 3 (IRF-3), facilitating its nuclear translocation and promoting the expression of IFN-α/β, thereby activating the antiviral immune response.^10^^,^^11^^,^^12^ Previous studies have demonstrated that overexpression of HO-1, either through small molecules or genetic approaches at the cellular or animal level, can inhibit viral replication by upregulating IFN-α/β without exacerbating the severity of infection.^5^^,^^11^ In fact, in the animal model, HO-1 overexpression suppressed the expression of inflammatory cytokines such as interleukin 6 (IL-6), IL-8, tumor necrosis factor alpha (TNF-α), and monocyte chemoattractant protein-1 (MCP-1), and mitigated lung injury in mice.^13^^,^^14^ The differential modulation of HO-1’s enzymatic activity and IRF3-inducing properties may contribute to its protective effects.^10^ These findings suggest that HO-1 may play a pivotal role in balancing the antiviral immune response with the prevention of excessive inflammatory damage.

In this study, we demonstrate that lung tropic adeno-associated virus (AAV) mediated overexpression of HO-1 significantly reduces lung damage and influenza virus replication. Mechanistically, HO-1 overexpression suppresses the excessive accumulation of plasmacytoid dendritic cells (pDCs) and classical monocytes in the lungs and spleen while increases frequency of Treg cells and nonclassical monocytes, thereby alleviating lung inflammation. The catalytically inactive mutation (H25A) disrupts HO-1’s ability to inhibit these inflammatory cell populations and reduces the frequency of Tregs and nonclassical monocytes. Additionally, HO-1 overexpression increases macrophage proportions, which enhance antiviral effects through upregulation of the type I IFN pathway. In HO-1 knockout mice, influenza virus infection is more severe, underscoring HO-1’s role in both lungs and spleen in controlling the virus. This study underscores HO-1’s crucial dual role in mediating antiviral immune responses and mitigating excessive inflammatory damage, with the H25A catalytic site being pivotal for its protective effect.

## Results

### HO-1 overexpression ameliorates lung tissue pathology and suppresses IAV replication in IAV-infected mice

**Figure 1:**
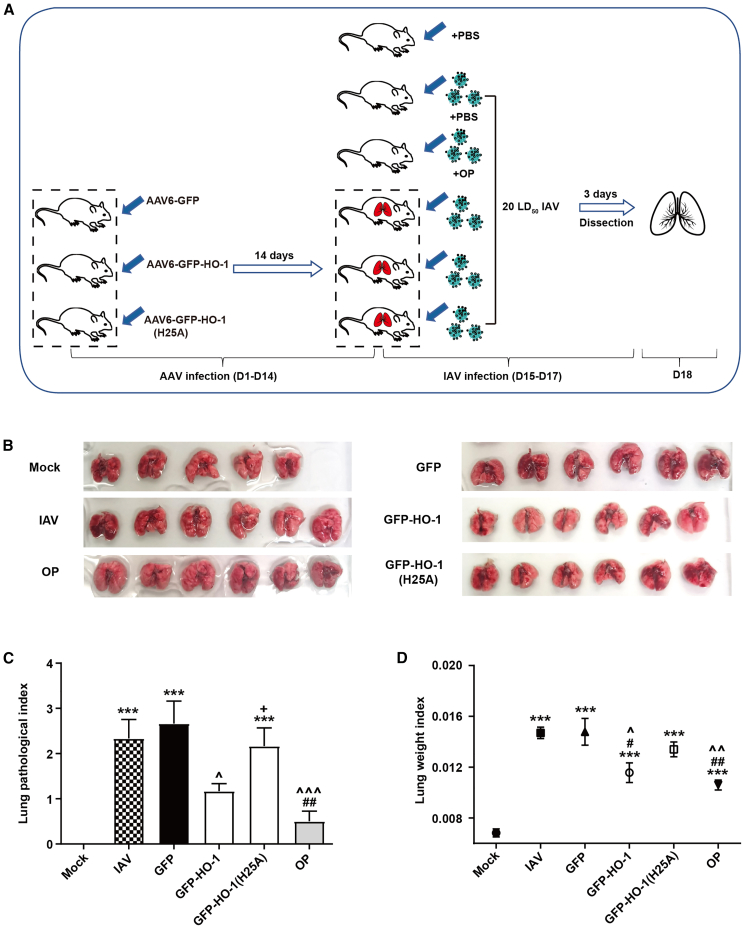
Lung tissue pathology of mice overexpressing wide-type and mutant HO-1 after infecting with influenza virus (A) Schedule of the AAV6-GFP-HO-1 and influenza virus administration. (B) Complete lung photos from mice. (C) Evaluation of lung pathological index. (D) Evaluation of lung weight index (lung weight/body weight). Mock: mice received intragastric gavage (i.g.) with PBS only; IAV: mice infected with A/FM1/1947-MA (FM1) at a dose of 20 LD_50_ and given i.g. with PBS; OP: IAV-infected mice treated with 15 mg/kg oseltamivir phosphate (OP) via i.g. administration; GFP, GFP-HO-1, GFP-HO-1(H25A): mice treated with AAV6-GFP, or AAV6-GFP-HO-1, or AAV6-GFP-HO-1(H25A) (2 × 10^11^ vg) for 14 days, followed by IAV infection (20 LD_50_). All mice used were Kunming mice. *n* = 5–6/group. Data were presented as mean ± SEM, ^∗∗∗^*p* < 0.001 versus mock; ^#^*p* < 0.05, ^##^*p* < 0.01 versus IAV; **ˆ***p* < 0.05, **ˆˆ***p* < 0.01, **ˆˆˆ***p* < 0.001 versus GFP; ^**+**^*p* < 0.05 versus OP.

Mice were administered AAV6 as a gene delivery vehicle to overexpress GFP-HO-1, GFP-HO-1(H25A), or GFP, designated as AAV6-GFP-HO-1, AAV6-GFP-HO-1(H25A), and AAV6-GFP. Fourteen days post-AAV6 inoculation, the mice were challenged with IAV with titers of 20 LD_50_. Lung tissues were collected 3 days after IAV infection to evaluate lung pathology, body weight, and lung weight. The entire experimental protocol is presented in Figure 1A. As shown in Figures 1B–1D, AAV6-GFP-HO-1 significantly reduced IAV-induced lung pathology compared to the AAV6-GFP group, as reflected by improvements of lung pathological index (Figure 1C) and lung weight index (lung weight/body weight) (Figure 1D), while AAV6-GFP-HO-1(H25A) treatment partially restored its lung pathology index and enhanced the lung weight index, compared with AAV6-GFP-HO-1 treatment, suggesting that the H25 site plays a critical role in mitigating lung damage.

**Figure 2:**
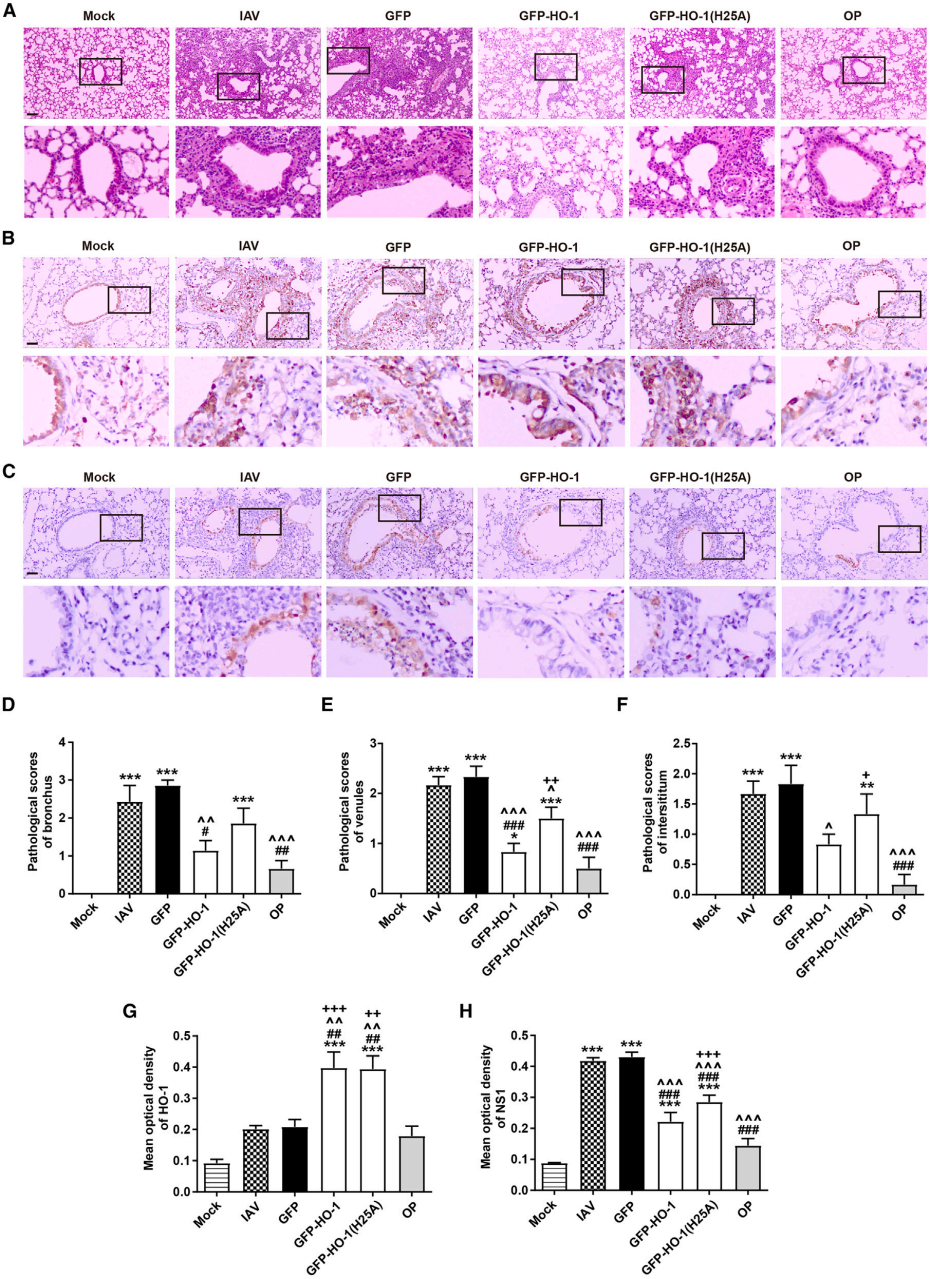
Pathological and immunohistochemical assessment of influenza-infected mice treated with AAV6-GFP-HO-1 and its mutant (A) H&E staining images of mouse lung tissue (scale bar, 20 μm). (B and C) Microscopic distribution of HO-1 (B) and influenza virus NS1 (C) (scale bar, 10 μm). (D–F) Pathological scores of the bronchi (D), small veins (E), and interstitium (F) in mouse lungs. (G and H) Quantitative analysis of mean optical density values for HO-1 (G) and NS1 (H) protein staining (*n* = 5–6/group). Values are presented as mean ± SEM. ^∗∗^*p* < 0.01, ^∗∗∗^*p* < 0.001 versus mock; ^#^*p* < 0.05, ^##^*p* < 0.01, ^###^*p* < 0.001 versus IAV; **ˆ***p* < 0.05, **ˆˆ***p* < 0.01, **ˆˆˆ***p* < 0.001 versus GFP; ^**+**^*p* < 0.05, ^**++**^*p* < 0.01, ^**+++**^*p* < 0.001 versus OP.

Meanwhile, the H&E staining results demonstrated that AAV6-GFP-HO-1 significantly mitigated lung tissue damage in influenza-infected mice on the third day post-infection compared to the AAV6-GFP group (Figure 2A). This was reflected in markedly lower inflammation scores in the bronchi, small veins, and alveolar interstitium, along with reduced areas of consolidation and significantly decreased inflammatory cell infiltration. The control drug, oseltamivir phosphate (OP), also effectively suppressed lung tissue damage at both time points assessed. However, the protective effect of AAV6-GFP-HO-1 against influenza-induced lung damage was attenuated in the AAV6-GFP-HO-1(H25A) group (Figures 2D–2F). These findings suggest that wild-type HO-1 AAV can reduce lung tissue damage in influenza-infected mice, while the H25A mutations compromise this protective effect.

Subsequent immunohistochemical analysis of lung tissues revealed that, 14 days post-AAV infection and 3 days post-influenza infection, both the IAV control group and the AAV6-GFP infection control group showed expression of the influenza virus structural protein NS1, along with a slight increase in HO-1 expression. In contrast, both wild-type AAV6-GFP-HO-1 and mutant AAV6-GFP-HO-1 treatment groups exhibited a significant upregulation of HO-1 expression (Figures 2B and 2G). Notably, NS1 protein expression in lung tissues was significantly suppressed following treatment with wild-type AAV6-GFP-HO-1 as well as mutant AAV6-GFP-HO-1(H25A), indicating that there was a significant negative correlation between HO-1 and NS1 in lung, Moreover, the H25A site does not appear to be crucial for the anti-IAV replication response (Figures 2C and 2H).

### HO-1 overexpression reduces the infiltration of inflammatory cells in the lungs and spleen, including pDCs and classical monocytes, following IAV infection

**Figure 3:**
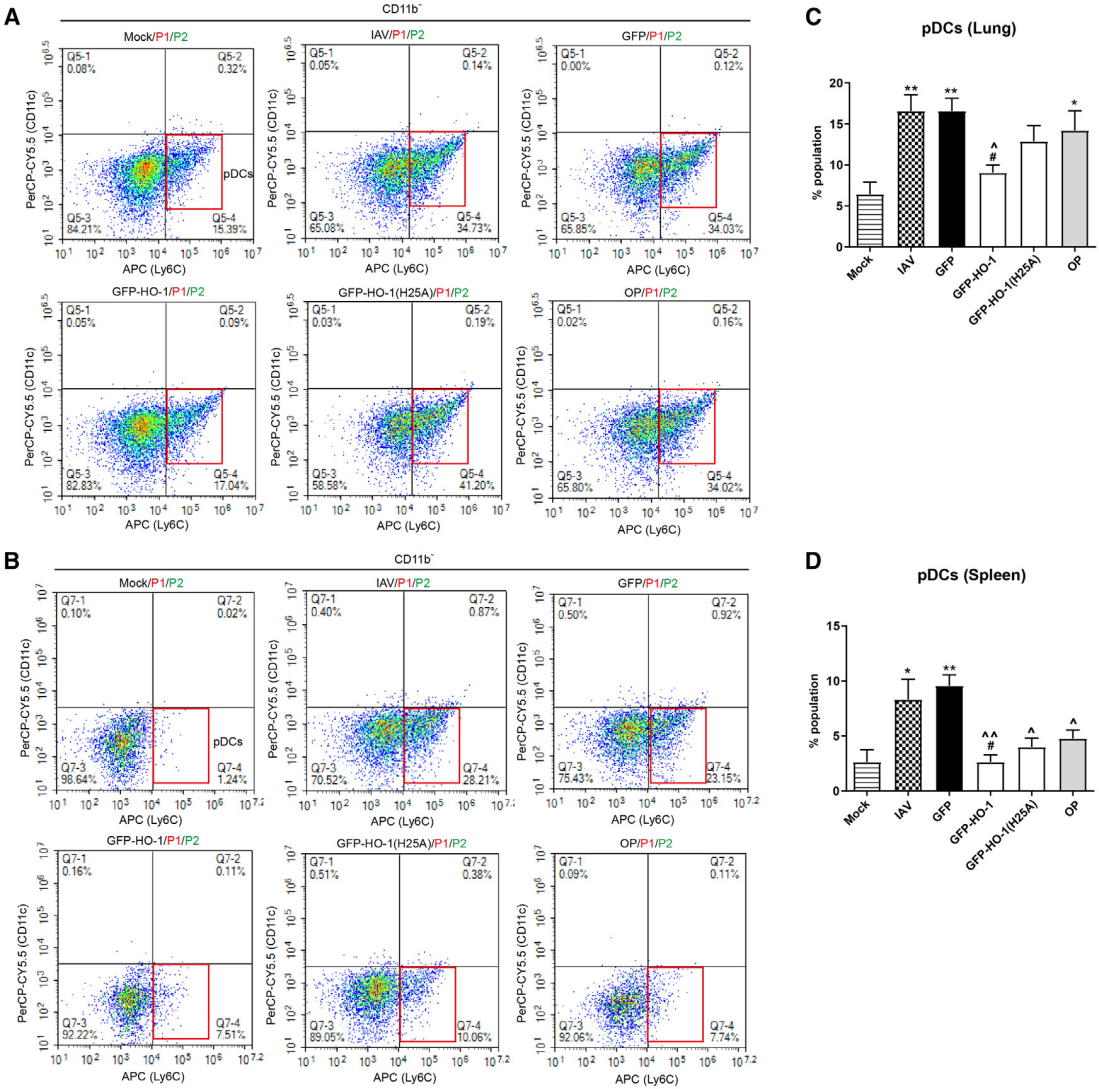
The impact of HO-1 and HO-1(25A) on lung-infiltrating pDCs and splenic pDCs (A) Flow cytometry analysis of the effect of wild-type and mutant HO-1 on the proportion of pDCs in the lungs. (B) Flow cytometry analysis of the effect of wild-type and mutant HO-1 on the proportion of pDCs in the spleen. (C and D) Quantitative analysis of pDCs proportions with respect to all live cells analyzed in the lungs (C) and spleen (D). *n* = 6/group, and tissues from 2 individual mice were pooled. Values are presented as mean ± SEM. ^∗^*p* < 0.05, ^∗∗^*p* < 0.01 versus mock; ^#^*p* < 0.05, ^##^*p* < 0.01, ^###^*p* < 0.001 versus IAV; **ˆ***p* < 0.05, **ˆˆ***p* < 0.01, **ˆˆˆ***p* < 0.001 versus GFP; ^**+**^*p* < 0.05, ^**++**^*p* < 0.01, ^**+++**^*p* < 0.001 versus OP. See also Figure S1.

**Figure 4:**
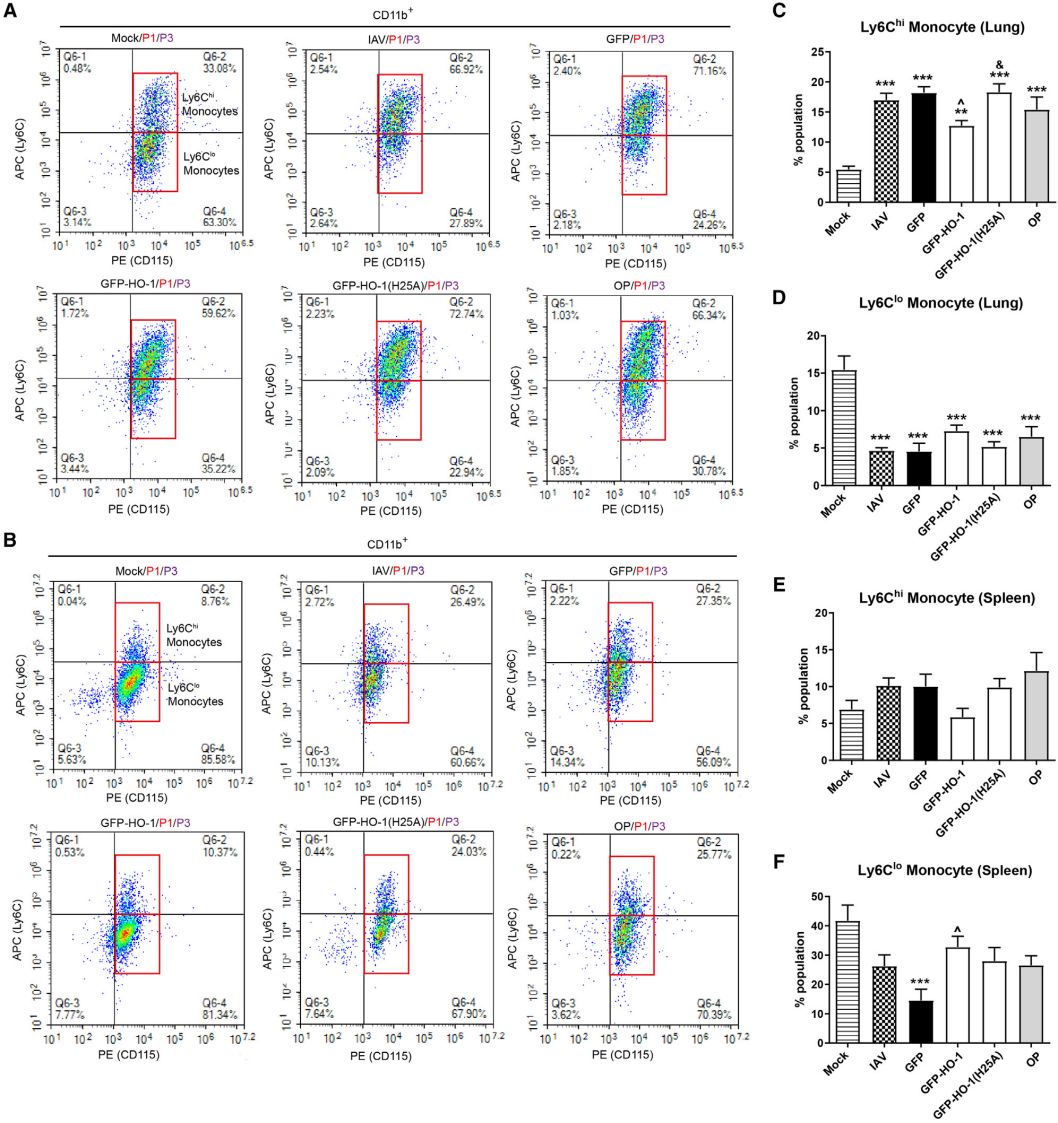
The impact of HO-1 and HO-1(25A) on lung-infiltrating monocytes and splenic monocyte (A and B) Flow cytometry analysis of the effect of wild-type and mutant HO-1 on the proportion of Ly6C^hi^ and Ly6C^lo^ monocytes in the lung (A) and the spleen (B). (C–F) Quantitative analysis of Ly6C^hi^ and Ly6C^lo^ monocyte proportions with respect to all live cells analyzed in the lung (C and D) and spleen (E and F). *n* = 6/group, and tissues from 2 individual mice were pooled. Values are presented as mean ± SEM. ^∗∗^*p* < 0.01, ^∗∗∗^*p* < 0.001 versus mock; **ˆ***p* < 0.05 versus GFP; ^&^*p* < 0.05 versus GFP-HO-1. See also Figure S1.

Mice were infected with AAV6-GFP-HO-1 or its mutant, followed by exposure to 20 LD_50_ of IAV. Lung tissue and spleen were collected 3 days post-IAV infection, and dissociated cells were analyzed by flow cytometry to evaluate the impact of HO-1 on inflammatory infiltrating cells, which play a crucial role in the overproduction of the pro-inflammatory cytokines. The fluorescence-activated cell sorting (FACS) tracings of different immune regulatory cells were displayed in Figure S1. As indicated in Figure 3, HO-1 overexpression significantly reduced the IAV-induced increase in pDCs (CD11c^low^/Ly6C^+^/CD11b^−^) in both the lungs (Figures 3A and 3C) and spleen (Figures 3B and 3D), compared to the AAV-GFP treatment group. Similarly, HO-1 overexpression also markedly suppressed the IAV-induced frequency of Ly6C^hi^ classical monocytes (CD11b^+^/CD115^+^/Ly6C^high^) in both the lungs (Figures 4A and 4C) and spleen (Figures 4B and 4E). Additionally, HO-1 overexpression increased the frequency of Ly6C^lo^ nonclassical monocytes (CD11b^+^/CD115^+^/Ly6C^low^) in both tissues, with significant differences observed in the spleen (Figures 4A, 4B, 4D, and 4F). However, the H25A mutation partially abolished the inhibitory effect of HO-1 on the two types of infiltrating inflammatory cells, including pDC and Ly6C^hi^ classical monocytes, in both the lungs and spleen, while also partially inhibiting the induction of Ly6C^lo^ nonclassical monocytes (Figures 3 and 4).

### HO-1 overexpression upregulates lung-infiltrating and splenic macrophages and regulatory T cells following IAV infection

**Figure 5:**
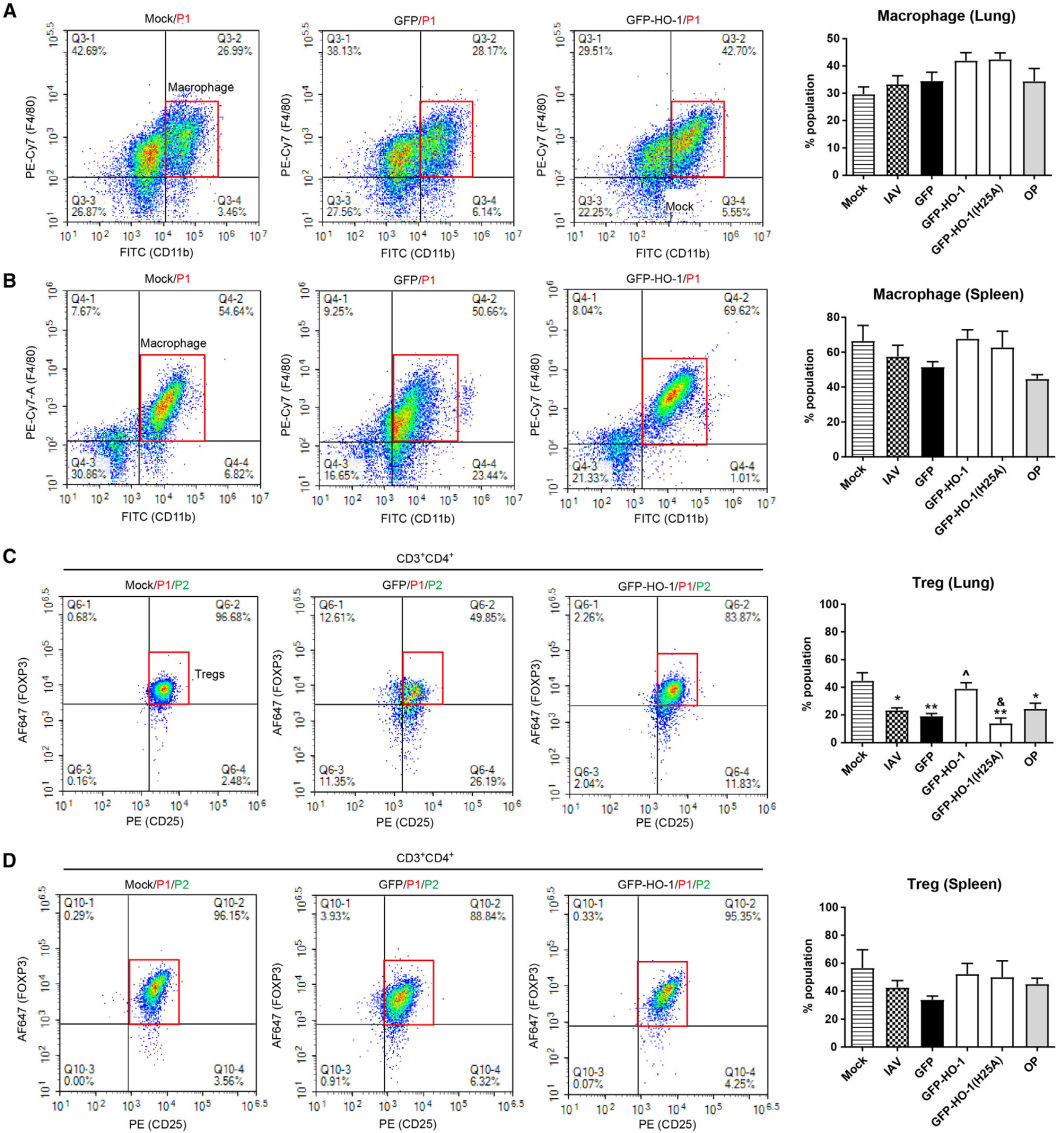
Impact of HO-1 and HO-1(H25A) on macrophage and Treg cell subsets in lung infiltrates and spleen (A and B) Flow cytometric analysis of the effects of wild-type and mutant HO-1 on macrophage proportions in the lung (A) and the spleen (B). (C and D) Flow cytometric analysis of the effects of wild-type and mutant HO-1 on Treg cell proportions with respect to all live cells analyzed in the lung (C) and the spleen (D). *n* = 6/group, and tissues from 2 individual mice were pooled. Values are presented as mean ± SEM. ^∗^*p* < 0.05, ^∗∗^*p* < 0.01 versus mock; ^**ˆ**^*p* < 0.05 versus GFP; ^&^*p* < 0.05 versus GFP-HO-1. See also Figure S1.

We next determined the effect of overexpressed HO-1 on lung-infiltrating and splenic macrophages. Flow cytometry analysis revealed that IAV infection led to a reduction in macrophages (CD11b^+^/F4/80^+^) in the spleen, accompanied by an increase in macrophage numbers in the lungs. This observation suggests a possible migration of macrophages from the spleen to the lungs post-IAV infection. Compared to the AAV6-GFP control group, AAV6-GFP-HO-1 overexpression resulted in a slight increased proportion of macrophages in both the lungs and spleen, whereas the H25A mutation did not significantly affect this enhancement (Figures 5A and 5B). Considering the critical role of macrophages in innate immunity within the respiratory tract and their involvement in lung defense against viral infections, we infer that HO-1 enhances anti-IAV immune responses by promoting the recruitment and activation of lung-infiltrating macrophages.

The study also demonstrated that IAV infection caused a decrease in regulatory T cells (Tregs, CD4^+^/CD25^+^/FOXP3) in both the lungs and spleen. However, the proportion of Tregs was significantly elevated in the AAV6-GFP-HO-1 group compared to the AAV6-GFP group. Tregs are critical in maintaining immune balance during viral infections and limiting tissue damage. Thus, HO-1 may contribute to mitigating lung inflammation following influenza virus infection through the activation of Treg cells. The H25A mutation, however, led to a reduction in Treg levels, which is consistent with the observed worsening of lung inflammation (Figures 5C and 5D).

### HO-1 overexpression induces type I IFN-stimulated proteins and suppresses inflammatory cytokines in lung tissues following IAV inflection

**Figure 6:**
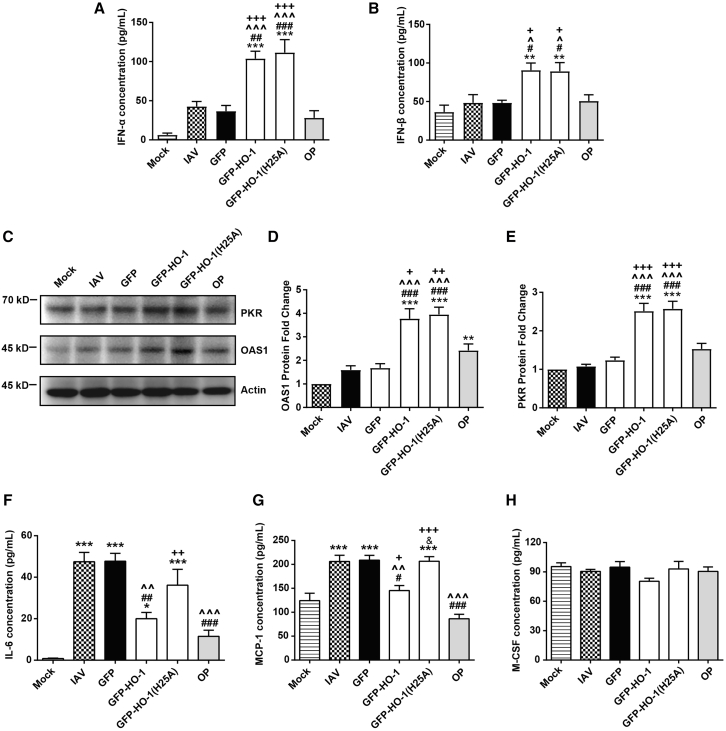
The effects of HO-1 and HO-1(25A) on the expression of interferon-induced proteins and inflammatory cytokines in lung tissue following influenza infection (A and B) ELISA analysis of the effects of wild-type and mutant HO-1 on the expression of IFN-α (A) and IFN-β (B) in serum. (C) Western blot analysis of the impact of wild-type and mutant HO-1 on the expression of PKR and OAS1 proteins. (D and E) Statistical analysis of OAS1 and PKR protein expression. (F–H) ELISA analysis of the effects of wild-type and mutant HO-1 on the expression of inflammatory cytokines in serum, including IL-6 (F), MCP-1 (G), and GM-CSF (H). *n* = 6/group. Values are presented as mean ± SEM. ^∗^*p* < 0.05, ^∗∗^*p* < 0.01, ^∗∗∗^*p* < 0.001 versus mock; ^#^*p* < 0.05, ^##^*p* < 0.01, ^###^*p* < 0.001 versus IAV; ^**ˆ**^*p* < 0.05, ^**ˆˆ**^*p* < 0.01, ^**ˆˆˆ**^*p* < 0.001 versus GFP; ^**+**^*p* < 0.05, ^**++**^*p* < 0.01, ^**+++**^*p* < 0.001 versus OP; ^&^*p* < 0.05 versus GFP-HO-1.

Given the observed upregulation of macrophages and Treg cells driven by elevated HO-1 expression, we further explored whether AAV6-GFP-HO-1 induces type I IFN-mediated innate immunity and suppresses pulmonary inflammation. Our results demonstrated that AAV-HO-1 significantly promoted the production of type I IFNs, including IFN-α and IFN-β in serum (Figures 6A and 6B). Western blot analyses revealed that AAV6-GFP-HO-1 enhances the expression of IFN-stimulated proteins in lung tissue, including OAS1 and PKR. Notably, the H25A mutation did not result in significant alterations in these expressions (Figures 6C–6E). Furthermore, ELISA results demonstrated that AAV6-GFP-HO-1 effectively inhibited the production of pro-inflammatory cytokines in lung tissue following influenza infection, specifically IL-6, MCP-1, and granulocyte-macrophage colony-stimulating factor (GM-CSF). The suppression of IL-6 and MCP-1 was statistically significant, with a diminished effect observed after the H25A mutation (Figures 6F–6H). These findings suggest that HO-1 overexpression may enhance macrophage activity, thereby activating the type I IFN pathway to confer antiviral protection. Additionally, HO-1 appears to upregulate Tregs and Ly6C^lo^ nonclassical monocytes while downregulating pDCs and Ly6C^hi^ classical monocytes, thus mitigating the excessive production of inflammatory cytokines. The anti-inflammatory properties of HO-1 are compromised by the H25A mutation.

### Conditional knockout of HO-1 gene exacerbates IAV infection in mice

To investigate the *in vivo* effects of HO-1 knockout on IAV infection in mice, we crossed HO-1 floxed homozygous/heterozygous mice with myeloid cell-specific Cre mice (Lyz2-Cre) and alveolar type II epithelial cell-specific Cre mice (Sftpc-CreERT2). This breeding produced myeloid-specific HO-1 knockout mice (HO-1 [flox/flox, Lyz2-Cre]) and lung epithelial cell-specific HO-1 knockout mice (HO-1 [flox/flox, Sftpc-CreERT2]). As demonstrated in Figure S2, we successfully generated HO-1 [flox/flox, Lyz2-Cre] and HO-1 [flox/flox, Sftpc-CreERT2] mice.

**Figure 7:**
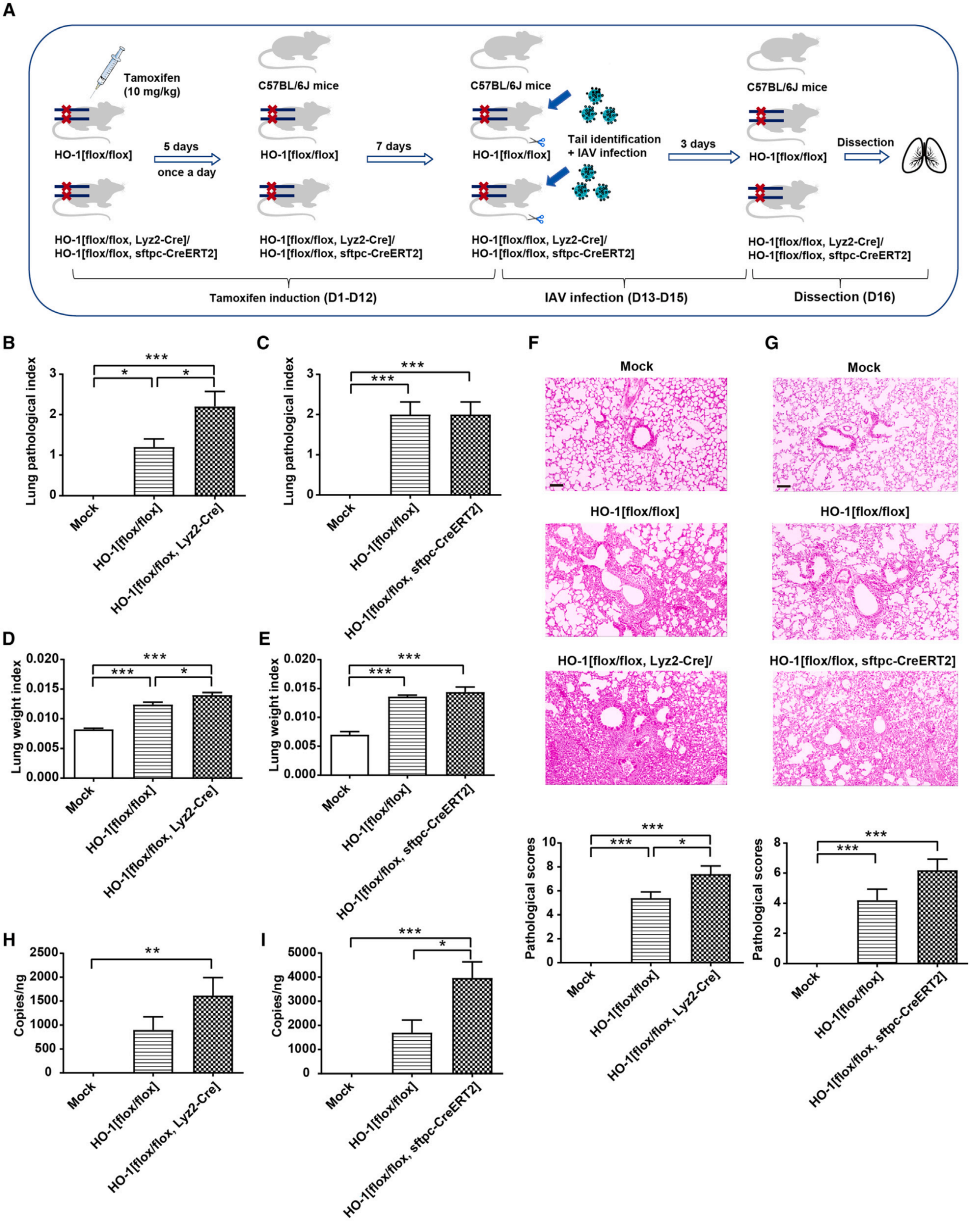
Effects of HO-1 gene knockout in myeloid and lung epithelial cells on lung tissue injury and viral load in IAV-infected mice (A) Schematic overview of the knockout mouse induction and IAV infection procedure. (B and C) Impact on lung pathological index in HO-1 conditional knockout mice. (D and E) Impact on lung weight index (lung weight/body weight) in HO-1 conditional knockout mice. (F and G) Assessment of lung tissue injury in HO-1 conditional knockout mice via H&E staining, with corresponding pathological scoring displayed (scale bar, 20 μm). (H and I) Effect on viral load in IAV infected HO-1 conditional knockout mice. *n* = 5/group. Values are presented as mean ± SEM. ^∗^*p* < 0.05, ^∗∗^*p* < 0.01, ^∗∗∗^*p* < 0.001. See also Figure S2.

Subsequently, we infected HO-1[flox/flox] mice and HO-1[flox/flox, Lyz2-Cre]/HO-1[flox/flox, Sftpc-CreERT2] mice with the H1N1 influenza virus, as depicted in Figure 7A. The results demonstrated that, compared to HO-1[flox/flox] mice, IAV infection caused more severe lung pathology index (Figures 7B and 7C) or increased lung weight index (lung weight/body weight) (Figures 7D and 7E) in both lung epithelial-specific HO-1 knockout mice and myeloid-specific HO-1 knockout mice. Notably, HO-1[flox/flox, Lyz2-Cre] mice exhibited more pronounced pathological damage (Figures 7B, 7C, 7F, and 7G). Further analysis revealed higher viral loads in the knockout mice compared to HO-1[flox/flox] controls, with a particularly significant increase in the HO-1[flox/flox, Sftpc-CreERT2] mice (Figures 7H and 7I). These findings highlight the critical role of HO-1 in myeloid and lung epithelial cells in defending against IAV infection and reducing lung tissue damage.

### Dual role of HO-1 on inhibiting IAV replication and alleviating pulmonary inflammation in lung epithelial cells-specific HO-1 knockout mice

**Figure 8:**
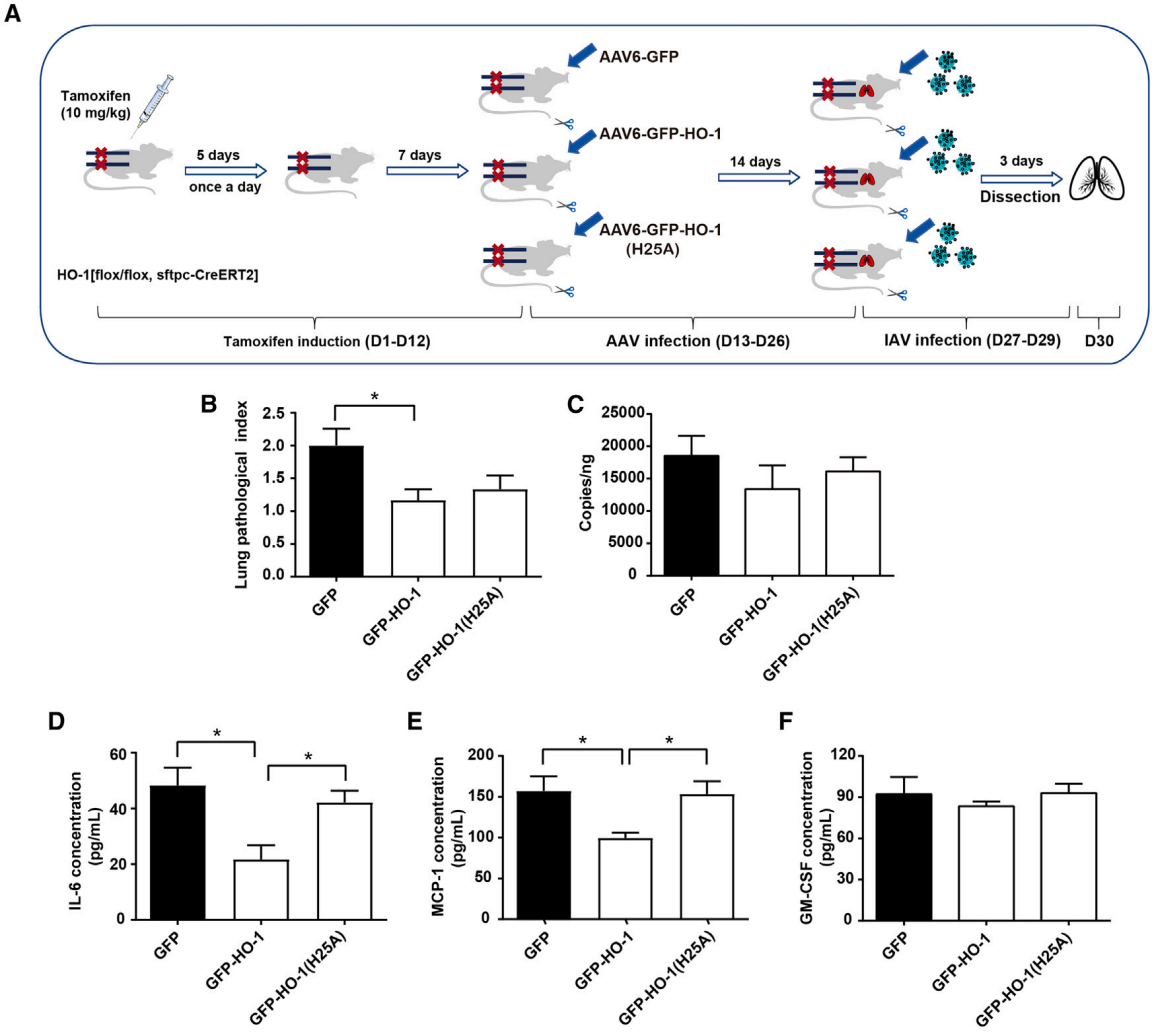
Impact of HO-1 and the HO-1(25A) mutant on IAV replication and pulmonary inflammation in HO-1[flox/flox, Sftpc-CreERT2] knockout mice (A) Schematic overview of the knockout mouse induction, AAV-HO-1 administration and IAV infection procedure. (B) Assessment of lung pathological index. (C) Quantification of IAV viral load using real-time RT-qPCR. (D–F) ELISA quantification of the effects of wild-type and mutant HO-1 on the expression of inflammatory mediators in lung tissue, including IL-6 (D), MCP-1 (E), and GM-CSF (F). *n* = 5/group. Values are presented as mean ± SEM. ^∗^*p* < 0.05.

To further explore the role of HO-1 and its enzymatic activity site mutation in IAV infection, we utilized lung epithelial cell-specific HO-1 knockout mice and divided them into three experimental groups. Each group was infected with AAV6-GFP, AAV6-GFP-HO-1, or AAV6-GFP-HO-1(H25A) for 14 days. Three days post-IAV infection, lung tissues were collected. The entire schedule was displayed in Figure 8A.

The results demonstrated that, compared to the AAV6-GFP group, AAV6-HO-1 significantly reduced IAV-induced lung lesions (Figure 8B) and moderately lowered influenza viral copy numbers in the conditional knockout mice (Figure 8C). Furthermore, AAV6-HO-1 effectively suppressed the overproduction of inflammatory cytokines including IL-6, MCP-1, and GM-CSF, with a statistically significant reduction observed for IL-6 and MCP-1 (Figures 8D–8F). In contrast, mutation of the H25A catalytic site resulted in partial increased lung pathological index, elevated viral copy numbers, and enhanced production of inflammatory cytokines. These findings suggest that the H25 site is critical for the protective function of HO-1 in lung tissue following IAV infection (Figures 8B–8F).

## Discussion

As a cytoprotective enzyme, HO-1 plays a crucial role in controlling various viral infections, making it a promising drug target for viral diseases. Studies have demonstrated that targeting HO-1 in viral infections can reduce the host’s inflammatory response, as seen in cases of HIV, Hepatitis B virus (HBV), Hepatitis C virus (HCV), and IAV.^15^^,^^16^^,^^17^^,^^18^^,^^19^ The ability of HO-1 to regulate the replication of certain viruses highlights its potential as an important agent against viral infections. However, the downregulation of inflammatory pathways may facilitate the intracellular growth of pathogens, raising the question of whether host HO-1 acts as a friend or foe in combating infections.^15^ Our previous work demonstrated that HO-1 exhibits anti-IAV infection effects by overexpression or pharmaceutical activation, primarily through the activation of the innate immune response.^5^^,^^11^ In this study, we propose a dual role of HO-1 in IAV infection, mediating antiviral immune responses while mitigating excessive inflammatory damage. It has been shown that HO-1 can effectively control viral replication without causing severe inflammatory damage, serving as a key host factor in achieving a balance between these two aspects.

pDCs and monocytes produce pro-inflammatory cytokines and activate immune effector cells such as neutrophils, CD8^+^ T cells, and NK cells, contributing to the clearance of IAV.^20^^,^^21^ However, the prolific cytokine production by pDCs during IAV infection can result in uncontrolled inflammation and host pathology,^8^ suggesting that pDCs may also play a role in the cytokine storm observed during infection. Similarly, excessive infiltration of monocytes can exacerbate IAV-induced host pathology.^22^^,^^23^ Moreover, during pregnancy, pDCs and monocytes exhibit a pronounced pro-inflammatory phenotype in response to IAV infection, increasing the risk of influenza-associated mortality.^24^ In our study, IAV infection led to an increased frequency of lung-infiltrating pDCs and classical monocytes as well as those in the spleen. HO-1, an important immune cell mediator,^25^ was found to downregulate the numbers of both pDCs and classical monocytes. Additionally, HO-1 increased the population of Tregs and nonclassical monocytes, which help protect the host from excessive inflammatory damage by maintaining immune system balance.^26^^,^^27^ Following AAV-GFP-HO-1 infection, pro-inflammatory cytokines such as IL-6, MCP-1, and GM-CSF were suppressed. Therefore, the primary mechanism by which HO-1 mitigates excessive lung inflammatory injury during IAV infection is by suppressing the pro-inflammatory response of pDCs and classical monocytes, meanwhile upregulating Treg and nonclassical monocytes population. The changes in immune cell numbers were dependent on the IAV dose and time, with significant effects observed at a high dose of 20 LD_50_ and 3 days after infection.

The protective effect of HO-1 has previously focused on its anti-inflammatory activity,^19^^,^^28^ and our team once made discovery revealing the anti-IAV role of HO-1 through both overexpression and pharmacological activation.^5^^,^^11^^,^^29^ In this study, we demonstrated that AAV-HO-1 infection in mice increased the macrophage population in both the lung and spleen to a certain extent, highlighting the important role of macrophage in HO-1-mediated anti-IAV response. Given previous finding that alveolar macrophages exert anti-IAV effects by upregulating type I IFN signaling pathways and inducing the expression of IFN-stimulated genes, thereby inhibiting viral spread,^30^^,^^31^ we infer that HO-1 may enhance the innate immune response to inhibit IAV replication through regulating macrophages. Notably, it has been reported that controlled activation of macrophages is critical for their protective role following influenza viral infections,^32^ which aligns with our observation that HO-1 slightly upregulated macrophage levels. Furthermore, we found that HO-1 could activate the type I IFN pathway to confer antiviral protection. Thus, we present evidence of the dual role of HO-1 in antiviral immune responses and in mitigating excessive inflammatory damage during influenza virus infection. Additionally, we observed more severe pathological damage and higher IAV viral loads in HO-1[flox/flox, Lyz2-Cre] and HO-1[flox/flox, Sftpc-CreERT2] knockout mice, indicating that HO-1 in both myeloid cell and lung epithelial cells is involved in defending against influenza infection and mitigating lung tissue damage. This study represents a preliminary exploration of HO-1’s effects on immune cell subpopulations, with the underlying mechanisms requiring further investigation. For instance, future studies will include intracellular staining for IFN-α and IFN-β in macrophages, monocytes and DCs to further elucidate the mechanisms involved.

The H25 site is a key catalytic site in heme metabolism, playing an essential role in the production of downstream metabolites.^33^^,^^34^ Mutation at the H25 site (H25A) has been shown to reduce lung damage, and largely reverse the effects of HO-1 on pDCs, monocytes and Tregs. Additionally, the H25A mutation alleviates the inhibition of HO-1 on the overproduction of inflammatory cytokines such as IL-6, MCP-1, and GM-CSF, while having no significant impact on the expression of ISGs like OAS1 and PRK. These findings suggest that the H25 site of HO-1 is specifically important for suppressing inflammatory injury rather than regulating the innate immune response. This aligns with previous observations that HO-1 induction by YZH-106 enhances the antiviral IFN response, potentially in a manner independent of HO-1’s enzymatic activity.^5^ Since IRF-3 directly interacts with HO-1 to induce the type I IFN response, future studies should focus on identifying the specific binding site between HO-1 and IRF-3. This could help clarify whether HO-1 regulates the innate immune response through a different mechanism than that involving the H25 site.

The induced expression of HO-1 can enhance the host’s antiviral immune response while simultaneously reducing inflammatory damage caused by the immune response. This discovery represents a significant advancement in understanding how the body balances the antiviral effects of the immune system with the risk of excessive inflammatory damage. In addition, individuals with different activities of the HO-1 gene promoter may show varying levels of HO-1 expression following virus infection, such as HIV, HCV, and COVID-19, indicating a clinical polymorphism in the HO-1 gene.^35^ Therefore, HO-1 may be a host factor influencing the severity of influenza. People with different activity of HO-1 gene promoter may show different expression levels of HO-1 after influenza infection, and determine the severity of influenza infection to a certain extent. Identifying polymorphisms in the HO-1 gene promoter to determine the level of HO-1 expression could be valuable in predicting which patients are at a higher risk of developing severe influenza, thereby enabling more precise prevention and treatment strategies. For instance, certain variants of the HO-1 gene with low transcriptional activity may be associated with increased susceptibility to IAV infections. In these cases, activating HO-1 through pharmaceutical activators or restoring its function via HO-1 gene therapy may help restore normal immune function and mitigate tissue damage caused by excessive inflammation.

### Limitations of the study

While the findings highlight the potential role of HO-1 in mitigating excessive inflammatory responses and enhancing antiviral immunity during IAV infection, the precise mechanisms by which HO-1 modulates specific immune cell populations, such as pDCs, monocytes, macrophages, and Tregs, remain incompletely understood and require further investigation. Moreover, this study predominantly addresses the effects of HO-1 overexpression, with less emphasis on the impact of HO-1 deficiency across various immune cell types, representing an area that warrants additional exploration.

## Resource availability

### Lead contact

Further information and requests for resources and reagents should be directed to and will be fulfilled by the lead contact, Yuhuan Li (yuhuanlibj@126.com).

### Materials availability

This study did not generate new unique reagents.

### Data and code availability

• This paper does not report original code.
• All data reported in this article will be shared by the lead contact upon request.
• Any additional information required to reanalyze the data reported in this paper is available from the lead contact upon request.

## Acknowledgments

The study was supported by the 10.13039/501100001809National Natural Science Foundation of China (grant no. 81902052 and 82394464 [82394460]) and 10.13039/501100019018CAMS Initiative for Innovative Medicine (grant no. 2021-I2M-1-030).

## Author contributions

Conceptualization and supervision, Y.L. and J.J.; methodology, investigation, formal analysis, and writing – original, L.M. and P.Z; investigation and data curation, X.L. and B.S.; funding acquisition, L.M, Y.L., and J.J.

## Declaration of interests

The authors declare no competing interests.

## STAR★Methods

### Key resources table

**Table:** 

REAGENT or RESOURCE	SOURCE	IDENTIFIER
**Antibodies**		

APC anti-mouse Ly6C	Biolegend	Cat# 128016; RRID: AB_1732076
PE/Cy7 anti-mouse F4/80	Biolegend	Cat# 123114; RRID: AB_893478
PE anti-mouse CD115 (CSF-1R)	Biolegend	Cat# 135506; RRID: AB_1937253
PerCP/Cyanine 5.5 anti-mouse CD4	Biolegend	Cat# 100434; RRID: AB_893324
PE anti-mouse CD25	Biolegend	Cat# 102008; RRID: AB_312857
Alexa Fluor® 647 anti-mouse/rat/human FOXP3	Biolegend	Cat# 320014; RRID: AB_439750
OAS1 rabbit polyAb	Proteintech	Cat# 14955-1-AP; RRID: AB_2158292
Anti-Heme Oxygenase 1 antibody	Abcam	Cat# ab13248; RRID: AB_2118663
β-actin Mouse mAb	Cell Signaling Technology	Cat# 3700; RRID: AB_2242334
PRK mouse monoclonal IgG	Santa Cruz Biotechnology	Cat# sc-6282; RRID: AB_628150
Influenza A ns1 mouse monoclonal IgG	Santa Cruz Biotechnology	Cat# sc-130568; RRID: AB_2011757
m-IgGκ BP-HRP	Santa Cruz Biotechnology	Cat# sc-516102; RRID: AB_2687626

**Bacterial and virus strains**		

A/FM1/1947-MA (FM1) influenza virus	This paper	N/A
AAV-GFP	WZ Biosciences Inc.	N/A
AAV-HO-1	WZ Biosciences Inc.	N/A
AAV-HO-1 (H25A)	WZ Biosciences Inc.	N/A

**Critical commercial assays**		

Lung Dissociation Kit, mouse	Miltenyi Biotec	Cat# 130-095-927
Spleen Dissociation Kit, mouse	Miltenyi Biotec	Cat# 130-095-926
True-Nuclear™ Transcription Factor Buffer Set	Biolegend	Cat# 424401
TruStain fcX™	Biolegend	Cat# 101320
T-PER Tissue Protein Extraction Reagent	Thermo Fisher Scientific	Cat# 78510
Halt Protease Inhibitor Cocktail	Thermo Fisher Scientific	Cat# 78430
BCA Protein Assay Kit	Thermo Fisher Scientific	Cat# 23227
ECL detection kit	Epizyme	Cat# SQ201
Mouse IL-6 ELISA kit	Absin	Cat# abs520004-96T
Mouse CCL2/JE/MCP-1 ELISA Kit	Absin	Cat# abs520016-96T
Mouse GM-CSF ELISA kit	Absin	Cat# abs520015-96T
Trizol Reagent	Invitrogen	Cat# 15596026CN
TransScript® II Green One-Step qRT-PCR SuperMix	Transgen	Cat# AQ311-01

**Experimental models: Organisms/strains**		

Kun Ming mice	Beijing HFK Bioscience Co. Ltd.	Cat# 12001A
HO-1 floxed mice	Institute of Laboratory Animal Sciences, Chinese Academy of Medical Sciences.	N/A
Lyz2-Cre mice	Cyagen Biosciences	Cat# C001358
Sftpc-CreERT2 mice	Cyagen Biosciences	Cat# C001359

**Software and algorithms**		

GraphPad Prism 8.0	GraphPad	https://www.graphpad.com/
FlowJo version 10	BD	https://www.flowjo.com/
Gelpro32	Media Cybernetics, Inc.	https://mediacy.com/

### Experimental model and study participant details

#### Ethics approval

The animal experiments were approved by the Animal Ethics Committee of Institute of Medicinal Biotechnology, Chinese Academy of Medical Sciences and Peking Union Medical College (Approval No.: IMB-20211202-D11-001; IMB-20211209-D11-001; IMB-20211225-D11-001).

#### Mouse infection

Female Kun Ming mice, aged 6-8-week-old and weighing approximately 14-16 g, were purchased from Beijing HFK Bioscience Co. Ltd. (Beijing, China). The mice were housed under controlled environmental conditions: a temperature of 22°C±1°C, relative humidity of 40% to 70%, and an ammonia concentration below 14 mg/m^3^, with a 12-hour light/dark cycle. The mice had free access to food and water.

Following 2-3 days of adaptive period, mice were randomly divided into the following experimental groups: Mock group (PBS), IAV group (PBS+IAV), AAV6-GFP control group (AAV-GFP+IAV), AAV6-GFP-HO-1 group, AAV6-GFP-HO-1(H25A) group, and oseltamivir phosphate (OP) treatment group (15 mg/kg). Adeno-associated viruses (AAV6-GFP, AAV6-GFP-HO-1, and AAV6-GFP-HO-1(H25A) were obtained from WZ Biosciences Inc. (Shandong, China) and were standardized to a concentration of 5×10^12^ vg/ml. Each mouse was intranasally administered 2×10^11^ vg in a 40 μl volume, with the infection model established over 14 days. Subsequently, the mice were intranasally infected with A/FM1/1947-MA (FM1) influenza virus at a dose of 20 LD_50_. Three days post-IAV infection, the mice were euthanized for the assessment of lung pathological indices and lung weight indices, as well as for pathological and immunohistochemical analyses. All Mice infection experiments were performed under ABSL-2 conditions and all animal procedures were conducted according to the standard operating procedures. The animal experiments were approved by the Animal Ethics Committee of Institute of Medicinal Biotechnology, Chinese Academy of Medical Sciences and Peking Union Medical College.

#### Generation of conditional HO-1 knockout mice

Conditional HO-1 knockout mice were established by generating HO-1 floxed gene mice at the Institute of Laboratory Animal Sciences, Chinese Academy of Medical Sciences. These HO-1 floxed mice were then crossed with Cre-expressing mice to produce tissue-specific knockouts for HO-1 in lung epithelial cells and myeloid cells. Specifically, HO-1 floxed homozygous/heterozygous mice were bred with myeloid cell-specific Cre mice (Lyz2-Cre) and alveolar type II epithelial cell-specific Cre mice (Sftpc-CreERT2) to obtain (flox/+, Lyz2-Cre) and (flox/+, Sftpc-CreERT2) conditional knockout (CKO) mice. Male (flox/flox, Lyz2-Cre) and (flox/flox, Sftpc-CreERT2) CKO mice derived from these breeding schemes were further crossed with HO-1 floxed homozygous females, resulting in HO-1 myeloid cell-specific knockout (HO-1 [flox/flox, Lyz2-Cre]) mice and HO-1 lung epithelial cell-specific knockout (HO-1 [flox/flox, Sftpc-CreERT2]) mice. The following primers were used for genotyping the mice: Hmox1-Loxp PCR: Forward (F1): 5’-TTGAGCATAGTGGTTGGAC-3’, Reverse (R1): 5’-TGGAGGAGGAGAGGAGAA-3’ (Product size: Homozygotes: 288 bp; Heterozygotes: 288 bp and 254 bp; Wildtype allele: 254 bp). Lyz2-Cre PCR: Forward (F2): 5’-CCCAGAAATGCCAGATTACG-3’, Reverse (R2): 5’-CTTGGGCTGCCAGAATTTCTC-3’ (Product size: ∼700 bp). Sftpc-CreERT2 PCR: Forward (F3): 5’-TGCTTCACAGGGTCGGTAG-3’, Reverse (R3): 5’-ACACCGGCCTTATTCCAAG-3’ (Product size: ∼210 bp).

### Method details

#### Evaluation of the lung pathological index and lung weight index in mice

Three days post-IAV infection, the mice were dissected, and their lungs were removed and rinsed with saline solution to assess the lung index. The severity of lung pathological index was evaluated based on a 5-grade scoring system: 0: Lung tissue appears white or light red, indicating no injury. 1: A few small, dark red inflammatory lesions are observed, but most of the lung tissue remains intact and lesion-free. 2: The inflammatory response has expanded, with dense clusters of dark red lesions. 3: Lung tissue damage has further expanded, covering the entire lung, accompanied by widespread diffuse inflammatory lesions and small areas of black necrotic tissue. 4: Extensive black necrosis is observed throughout the entire lung tissue, along with severe inflammatory lesions. Additionally, the lung weight was measured, and the lung weight index was calculated as the ratio of lung weight to body weight.

#### Lung histological assessment and immunohistochemistry (IHC)

Lung tissue sections were fixed in neutral buffered formalin (NBF) and stained with hematoxylin and eosin (H&E). IHC was performed using antibodies against IAV NS1 (Santa Cruz, Dallas, Texas, USA) and HO-1 (Abcam, Cambridge, MA, USA). All images were assessed by the pathologist in a blind manner. The pathological scoring of lung bronchus, venules and interstitium was based on the following criteria: 0: No inflammatory cell infiltration and no Interstitial pneumonia; 1: Mild inflammatory cell infiltration around the bronchus and venules, with mild hyperplasia of the alveolar septal; 2: Moderate inflammatory cell infiltration with fewer than 5 cell layers, thickened alveolar septal, and narrowed alveolar cavity; 3, severe inflammatory cell infiltration with more than 5 cell layers, large patchy areas of interstitial pneumonia consisting of hypercellular septa, significantly narrowed alveolar cavity, and a large amount of exudation. The intensity of IHC staining was quantified based on optical density (OD). Pathological scores per group (n=6) were used for statistical analysis of results.

#### Flow cytometry analysis

Mice were divided into 6 groups as previous described. They were infected with AAV6-GFP, AAV6-GFP-HO-1 or AAV6-GFP-HO-1(H25A) for 14 days, followed by exposure to 20 LD_50_ of IAV. Lung tissue and spleen were collected 3 days post-IAV infection and divided into two panels, and tissues were dissociated into single-cell suspension with the Lung Dissociation Kit and Spleen Dissociation Kit (Miltenyi Biotec, Bergisch Gladbach, Germany). Cells from first panel were pre-treated with an Fc receptor blocker (TruStain fcX™) (Biolegend, San Diego, CA) for 10 min to prevent non-specific binding of Fc receptors expressed on myeloid cells. The cells were then stained with APC anti-mouse Ly6C (Biolegend), PE/Cy7 anti-mouse F4/80 (Biolegend) and PE anti-mouse CD115 (CSF-1R) (Biolegend). Flow cytometry analysis was performed to identify cell subpopulations of pDCs (CD11c^low^/Ly6C^+^/CD11b^−^), Ly6C^hi^ monocytes (CD11b^+^/CD115^+^/Ly6C^high^), Ly6C^lo^ monocytes (CD11b^+^/CD115^+^/Ly6C^low^), and macrophages (CD11b^+^/F4/80^+^). Meanwhile, cells from the second panel were fixed with a True-Nuclear™ Transcription Factor Buffer Set (Biolegend) for 30 mins to permeabilize the membrane. Tregs (CD4+/CD25+/FOXP3) were stained with FITC anti-mouse CD3 (Biolegend), PerCP/Cyanine5.5 anti-mouse CD4 (Biolegend), PE anti-mouse CD25 (Biolegend) and Alexa Fluor® 647 anti-mouse/rat/human FOXP3 (Biolegend) and analyzed on the Novocyte Flow Cytometer (ACEA Biosciences, Inc., San Diego, CA, USA).

#### Western blotting analysis

For protein extraction from lung tissue, cells were lysed using T-PER Tissue Protein Extraction Reagent with Halt Protease Inhibitor Cocktail (Thermo Fisher Scientific, Waltham, MA, USA) and homogenized at 60 HZ for 120 s. Protein concentrations were determined using BCA Protein Assay Kit (Thermo Fisher Scientific). Equal amounts of proteins were subjected to SDS-PAGE and then transferred to PVDF membranes (Millipore, Billerica, MA, USA). After blocking with 5% milk, membranes were incubated with primary antibodies against OAS1 (Proteintech, Rosemont, IL, USA), PRK (Santa Cruz) and β-actin (Cell Signaling Technology, Beverly, MA, USA), respectively. Detection was performed by incubating with HRP-conjugated secondary antibodies (Santa Cruz) and an ECL detection kit (Epizyme, Shanghai, China). Signals were visualized using the Tanon 4000 imaging system (Tanon, China).

#### ELISA assay

Serum was collected by allowing blood to stand at room temperature for 30 minutes, followed by centrifugation at 1000 g for 15 minutes. Concentrations of inflammatory cytokines, including IL-6, MCP-1, and GM-CSF, were measured using ELISA kit (Absin, Shanghai, China) according to the manufacturer’s instructions. The absorbance was read at 450 nm with 570 nm as the corrected wavelength using an ELISA microplate reader (Aosheng, Hangzhou, China).

#### Viral quantitation

Total RNA was extracted from mouse lung tissue using Trizol Reagent (Invitrogen, Carlsbad, CA, USA), and its concentration was quantified. The PHW2000-PR8 M cDNA plasmid was utilized to generate a standard curve. The formula used for copy number calculation is: copies/μl = 6.02×10^23^ × 10^-9^ × concentration / (base number × 660). For the standard curve, serial 10-fold dilutions ranging from 3×10^9^ to 3×10^3^ copies/μl were prepared. Real-time PCR detection was performed using the TransScript® II Green One-Step qRT-PCR SuperMix (Transgen, Beijing, China). Primer sequences were as follows: IAV M Forward: 5'GACCRATCCTGTCACCTCTGAC3', Reverse: 5'GGGCATTYTGGACAAAKCGTCTACG3'. Copy numbers were quantified using the ABI 7500 Fast real-time PCR system (Applied Biosystems, Foster City, CA, USA).

### Quantification and statistical analysis

Statistical analyses were conducted using GraphPad Prism 8.0 software. The sample size (n), representing the number of animals, and the statistical significance levels are specified in the figure legends. Results are presented as mean ± SEM. Data were analyzed using one-way ANOVA with Holm-Sidak multiple comparisons test. Statistical significance is denoted by symbols (∗, #, **ˆ**, **+**, &) to indicate comparisons between different groups. For instance, significance levels defined as ^∗^*P* < 0.05, ^∗∗^*P* < 0.01, ^∗∗∗^*P* < 0.001 versus Mock.
