# Supplementary material for: Dual role of HO-1 in mediating antiviral immune responses and mitigating excessive inflammatory damage during influenza virus infection
Source: iScience. 2025 Feb 26;28(3):112116. doi: 10.1016/j.isci.2025.112116 (PMC11951048; doi:10.1016/j.isci.2025.112116)
Supplement: Document S1. Figures S1 and S2 [file mmc1.pdf]

iScience, Volume *28*

Supplemental information

Dual role of HO-1 in mediating antiviral immune responses and mitigating excessive inflammatory damage during influenza virus infection

Linlin Ma, Peng Zhang, Xingqiong Li, Baihe Sun, Yuhuan Li, and Jiandong Jiang

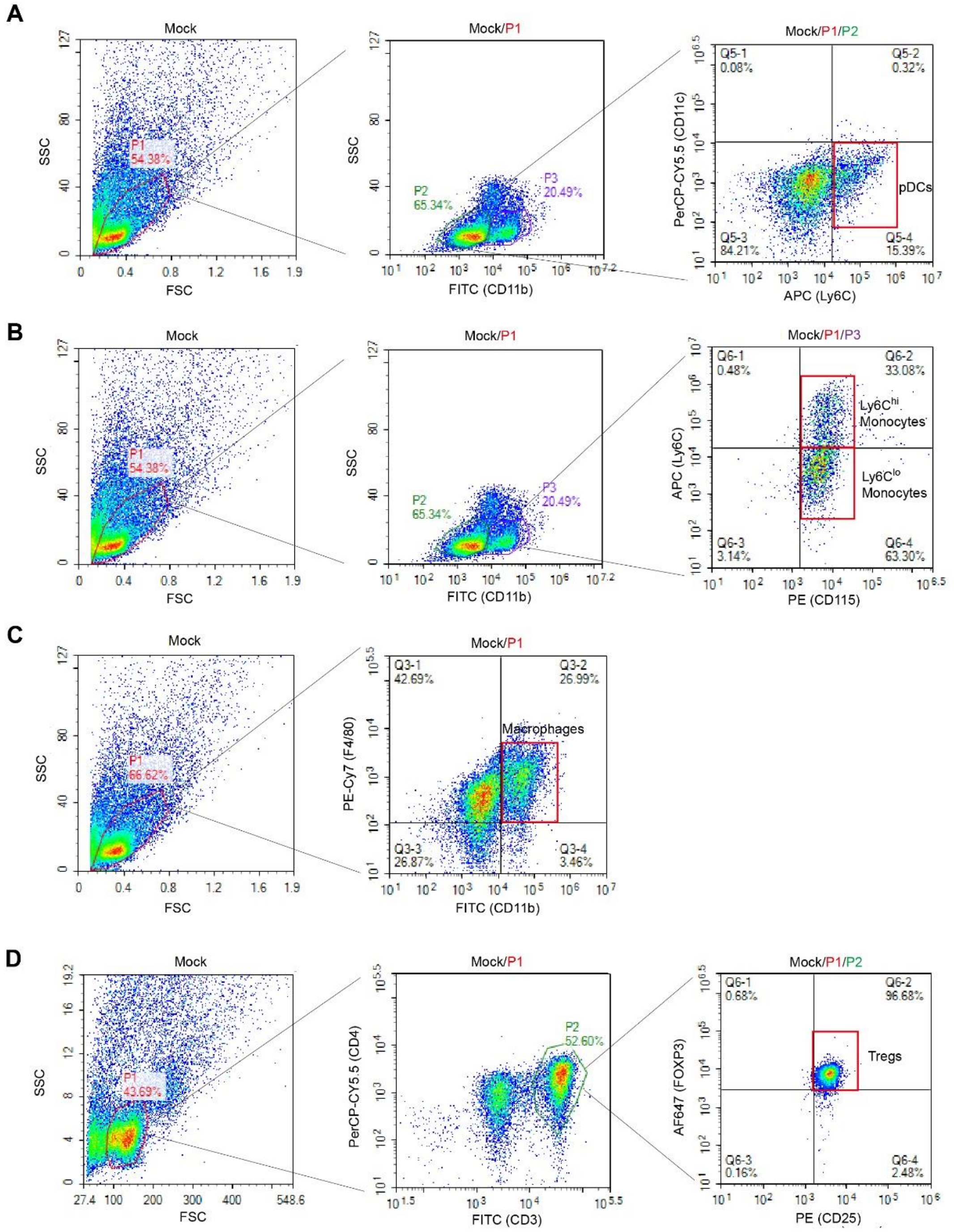

Figure S1. FACS tracings of different immunology regulatory cells, including pDCs (A), Ly6C^{hi} Monocytes, Ly6C^{lo} Monocytes (B), Macrophages (C) and Tregs (D). Related to Figures 3-5.

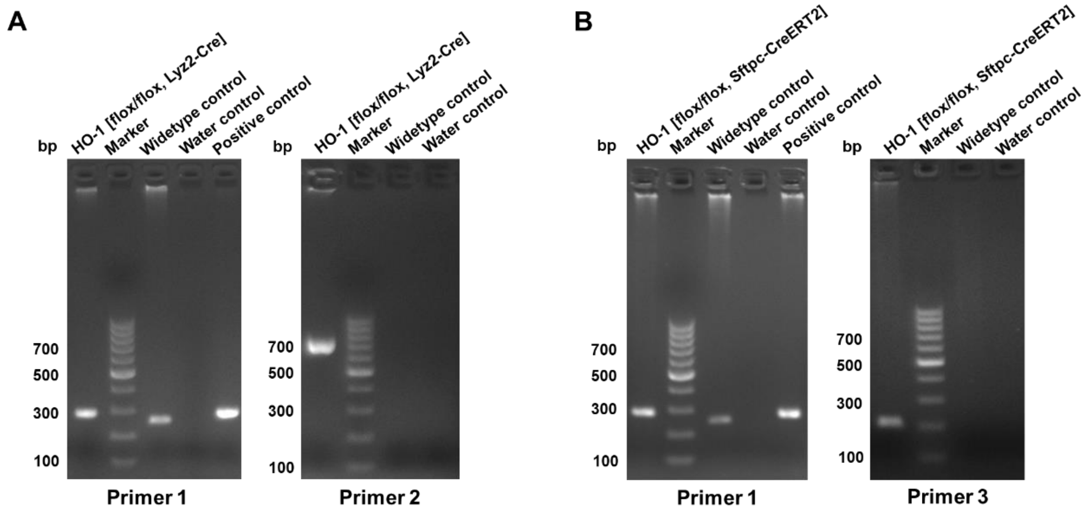

Figure S2. Identification of tissue-specific HO-1 gene knockout mice. Related to Figure 7. (A) Identification of HO-1 [flox/flox, Lyz2-Cre] mice. (B) Identification of HO-1 [flox/flox, Sftpc-CreERT2] mice. Primer 1: primers for HO-1-flox PCR; Primer 2: primers for Lyz2-Cre PCR; Primer 3: primers for Sftpc-CreERT2 PCR. Wildtype control: mouse genomic DNA. Water control: no DNA template added. Positive control: homozygotes positive mouse (HO-1 [flox/flox]) genomic DNA.
